# Proceedings of a membrane update symposium: advancements, scientific insights, and future trends for dialysis membranes for enhanced clinical outcomes in end stage kidney disease patients

**DOI:** 10.3389/fneph.2024.1455260

**Published:** 2024-10-15

**Authors:** Christoph Wanner, Raymond Vanholder, Alberto Ortiz, Andrew Davenport, Bernard Canaud, Peter J. Blankestijn, Rosalinde Masereeuw, Jeroen Peter Kooman, Giuseppe Castellano, Dimitrios Stamatialis, Sandip Mitra, Muriel Grooteman, Viktoria Weber, Thomas Ebert, Amira Abdelrasoul, Sonja Steppan, Anna Rebecca Scheiwe, Peter Stenvinkel

**Affiliations:** ^1^ Renal Research Unit, Department of Clinical Research and Epidemiology, University of Würzburg, Würzburg, Germany; ^2^ Clinical Trial Service and Epidemiology Studies Unit, Nuffield Department of Population Health, University of Oxford, Oxford, United Kingdom; ^3^ Nephrology Section, Department of Internal Medicine and Pediatrics, University Hospital, Ghent, Belgium; ^4^ European Kidney Health Alliance, Brussels, Belgium; ^5^ Department of Nephrology and Hypertension, IIS-Fundacion Jimenez Diaz UAM, Madrid, Spain; ^6^ Departamento de Medicina, Facultad de Medicina, Universidad Autónoma de Madrid, Madrid, Spain; ^7^ UCL Department of Nephrology, Royal Free Hospital, University College London, London, United Kingdom; ^8^ School of Medicine, University of Montpellier, Montpellier, France; ^9^ Department of Nephrology and Hypertension, University Medical Centre Utrecht, Utrecht University, Utrecht, Netherlands; ^10^ Division of Pharmacology, Utrecht Institute for Pharmaceutical Sciences, Utrecht University, Utrecht, Netherlands; ^11^ Division of Nephrology, Department of Internal Medicine, Maastricht University Medical Centre+, Maastricht, Netherlands; ^12^ Department of Internal Medicine, NUTRIM School of Nutrition and Translational Research in Metabolism, Maastricht University, Maastricht, Netherlands; ^13^ Department of Nephrology, Dialysis and Renal Transplantation, Fondazione IRCCS Ca’ Granda Ospedale Maggiore Policlinico, Milan, Italy; ^14^ Department of Clinical Sciences and Community Health, University of Milan, Milan, Italy; ^15^ Advanced Organ Bioengineering and Therapeutics-Technical Medical Centre, University of Twente, Enschede, Netherlands; ^16^ Department of Nephrology, Radboud University Medical Center, Radboud Institute for Molecular Life Sciences, Nijmegen, Netherlands; ^17^ Manchester Academy of Health Sciences Centre, Manchester University Hospitals, University of Manchester, Manchester, United Kingdom; ^18^ Department of Nephrology, Amsterdam UMC, location Vrije Universiteit Amsterdam, Nephrology, Amsterdam, Netherlands; ^19^ Department of Nephrology, Amsterdam Cardiovascular Sciences, Diabetes & Metabolism, Amsterdam, Netherlands; ^20^ Department for Biomedical Research, University for Continuing Education Krems, Krem, Austria; ^21^ Medical Department III - Endocrinology, Nephrology, Rheumatology, University of Leipzig Medical Center, Leipzig, Germany; ^22^ Division of Biomedical Engineering, Department of Chemical and Biological Engineering, University of Saskatchewan, Saskatoon, SK, Canada; ^23^ Fresenius SE & Co. KGaA, Bad Homburg, Germany; ^24^ Division of Renal Medicine, Department of Clinical Science, Intervention and Technology, Karolinska Institutet, Stockholm, Sweden

**Keywords:** dialysis membranes, innovation, biocompatibility, science, technology, clinical impact, chronic kidney disease

## Abstract

**Purpose of symposium:**

From September 6 – 8 2022, the Life/2022 Membrane Symposium was held in Frankfurt, Germany, and transmitted live to a worldwide internet audience. The event was part of the Life/Nephrology Campus initiative, a continuous educational platform for the nephrology community to expand knowledge and share expertise on contemporary topics in chronic kidney disease. We describe recent questions and advances in the field, and we underline challenges in the care of dialysis patients and opportunities for integration of new findings into clinical practice to improve patient outcomes in end stage kidney disease patients.

**Topics:**

Most patients with kidney failure are on maintenance hemodialysis (MHD). The scientific program of the symposium was developed around topics about the role, functional determinants, technical aspects, limitations, and clinical implications of membranes presently in use. International experts with clinical or technical expertise as well as scientific recognition within the nephrology community were asked to prepare their presentations based on their own experiences, perceptions, opinions, and sources of information. The symposium devoted a major portion to discussing novel approaches for improving membranes and treatment quality, including updates on innovative concepts that may could potentially transform the landscape of kidney replacement therapy for chronic kidney disease patients in the future.

**Implications:**

The intent was to provide insights into current attention points for healthcare professionals new to the field of MHD, and to test a unique forum for continuing medical education integrating physician and patient experiences to promote changes in clinical practice. Furthermore, the symposium premiered a specifically developed mixed reality holographic 3D model to demonstrate recent dialyzer innovation diminishing protein fouling on membrane surfaces. As a continuous online educational platform for scientific exchange, this Life/2022 event provided online learning opportunities with on-demand content, with all symposium lectures freely available on *nephrologycampus.com*.

## 1 Symposium context and objectives

The demand for MHD is projected to increase significantly over the next decade, increasing the burden for healthcare professionals (HCPs) and systems (1, 2). There’s a growing concern regarding the shortage of skilled nephrology professionals, which could impact the quality of dialysis care. Nephrology is a complex medical field due to the multifaceted nature of diseases, technological integration, and cost pressures. When nine different markers of patient complexity were evaluated, Nephrology was ranked as the most complex of all disciplines (3).

To address the reluctance in choosing nephrology as a career, more efficient and time-saving educational methods and utilization of modern online tools, have gained traction. The Life/nephrology campus strives to provide concise, updated educational material collated by experts for newcomers as well as for established MHD care personnel.

The Life/2022 Membrane Symposium focused on recent advances in membrane technology, particularly in uremic toxin removal in dialysis patients. Renowned experts discussed issues revolving around chronic inflammation and the role of dialysis in bioincompatibility. They also highlighted upcoming technical innovations promising improved patient outcomes and quality of life.

This symposium introduced a novel educational approach, combining on-site speaker presentations for healthcare professionals with a real-time online global audience. The content was categorized into six sections (**Table 1**), available for scientific discussion and continuous education on nephrologycampus.com. The online library also includes visual aids to enhance learning accessibility, such as movies and animated videos elucidating complex membrane science concepts, e.g., a movie explaining the stages of production of both membranes and dialyzers, and an animated video series depicting the membrane perspective of uremic toxins.

**Table 1:** Key topics of the Membrane Symposium: aspects of progress in dialysis membrane technology aiming for an improvement of clinical results and topics within the Membrane Symposium.

Key topics of the membrane symposium					
Delivery of hemodialysis	Chronic inflammation	Inflammation bioincompatibility axis	Blood-material interaction	Membrane and dialyzer technology	Emerging trends and future frontiers
**V**					
**Improving Clinical Outcomes and Therapy Tolerance**					

### 1.1 Delivery of hemodialysis

Patients with end-stage kidney disease (ESKD) undergo MHD with approximately 89% experiencing a substantial disease burden, reduced life expectancy, notable symptom burden, and a low health-related quality of life (2). These patients have a complex clinical profile, commonly presenting multiple comorbidities, especially cardiovascular (CV) pathologies, protein-energy wasting, and diabetes (4), with increasing age exacerbating their health complications (5). Albeit life-saving, clinical outcomes associated with MHD lag behind the general population and other chronic diseases (2). Rather than exclusively prioritizing survival, there’s an increased focus on symptom alleviation and enhancing functional and social rehabilitation (1). The current intermittent dialysis schedule hampers effective uremic solute removal, internal milieu restoration, clinical tolerance, and patient rehabilitation (6, 7). Research suggests more frequent and extended sessions, often referred to as intensive dialysis, could mitigate these challenges, yet concerns about elevated costs impede widespread adoption by payors and healthcare systems (1, 8). Technological advances in extracorporeal circuitry (ECC) have historically improved the efficiency, reliability, safety, and handling of renal replacement therapy procedures (9, 10). The dialysis membrane, while crucial for detoxification in MHD, also contributes to bioincompatible reactions with their entailed dialysis-related morbidity.

**Figure 1:**
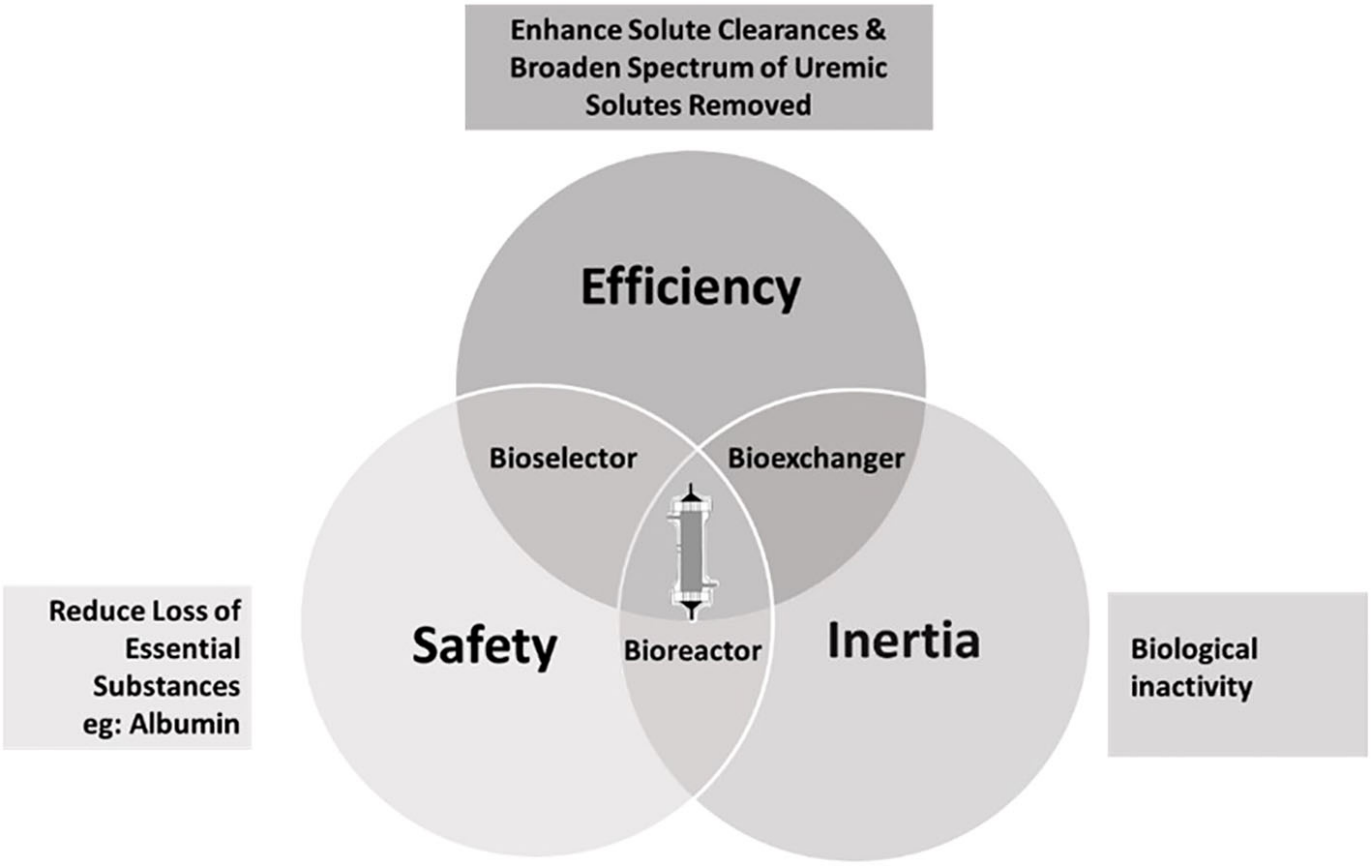
Relationships between the three most characteristic dialyzer functions. Modified from Canaud et al. (13).

MHD facilitates life prolongation by regularly reducing concentrations of harmful compounds (11). A semi-permeable membrane allows for size-dependent elimination of substances to combat the toxic uremic milieu. The membrane’s structure, dialyzer design, and operating conditions determine solute exchange efficiency (12). Each dialyzer/membrane type presents three interrelated functionalities, each of them decisive for therapeutic quality: a ‘bioexchanger,’ eliminating toxins and excess water, a ‘bioreactor,’ initiating biochemical reactions, and a ‘bioselector,’ balancing the removal of unwanted and essential substances (13) (**Figure 1**). Despite progress, advancements in membrane science and technology remain vital for refining dialysis therapies (14).

The assessment and removal of accumulated uremic retention solutes (URS) during MHD are fundamental for replacing kidney function in ESKD. Traditionally, URS classification focused on size (15), aiding membrane development but limiting clinicians’ ability to reduce circulating uremic toxin levels affecting patients’ well-being (2). A more goal-directed classification based on biological/chemical and clinical toxicity (16) has been proposed to grade key URS compounds (17) (**Table 2**), identify target molecules for removal, and develop novel elimination methods. However, distinguishing between toxic and beneficial metabolites remains challenging (18), as MHD follows size-exclusion, not toxicity principles. Hence, essential substances (*e.g.*, albumin) can leak through membranes with larger pore size (19), calling for a paradigm shift toward focusing on the dialysis-related loss of beneficial metabolites (20). The uremic milieu entails various biochemical disorders (*i.e.*, inflammation, oxidative and chlorine stresses, and carbamylation reactions) that modify accumulated compounds and increase their toxicity (*i.e.*, beta2-microglobulin and B2M-amyloidosis). Additionally, discussions often overlook the adverse effects of inorganic compounds like water, sodium, potassium, and phosphate (21). impacting patients’ quality of life (QoL) and contributing to hemodynamic instability, CV morbidity, and mortality in MHD patients (22). Tissue sodium accumulation contributes to cardiac and metabolic disorders independently from its osmotic and hemodynamic action (22). Hyperphosphatemia is important in the pathogenesis of secondary hyperparathyroidism, endothelial dysfunction (23), and increased all-cause and CV mortality (24). During MHD, considerable amounts of intracellular phosphate are removed as observed by a significant decrease in intracellular ATP, reflecting MHD-induced cellular stress and/or intracellular phosphate regulation with potentially severe clinical consequences (25, 26). Understanding and managing these compounds’ effects are essential in optimizing treatment strategies for patients undergoing dialysis.

**Table 2:** Uremic toxin classification based on evidenced toxicity.

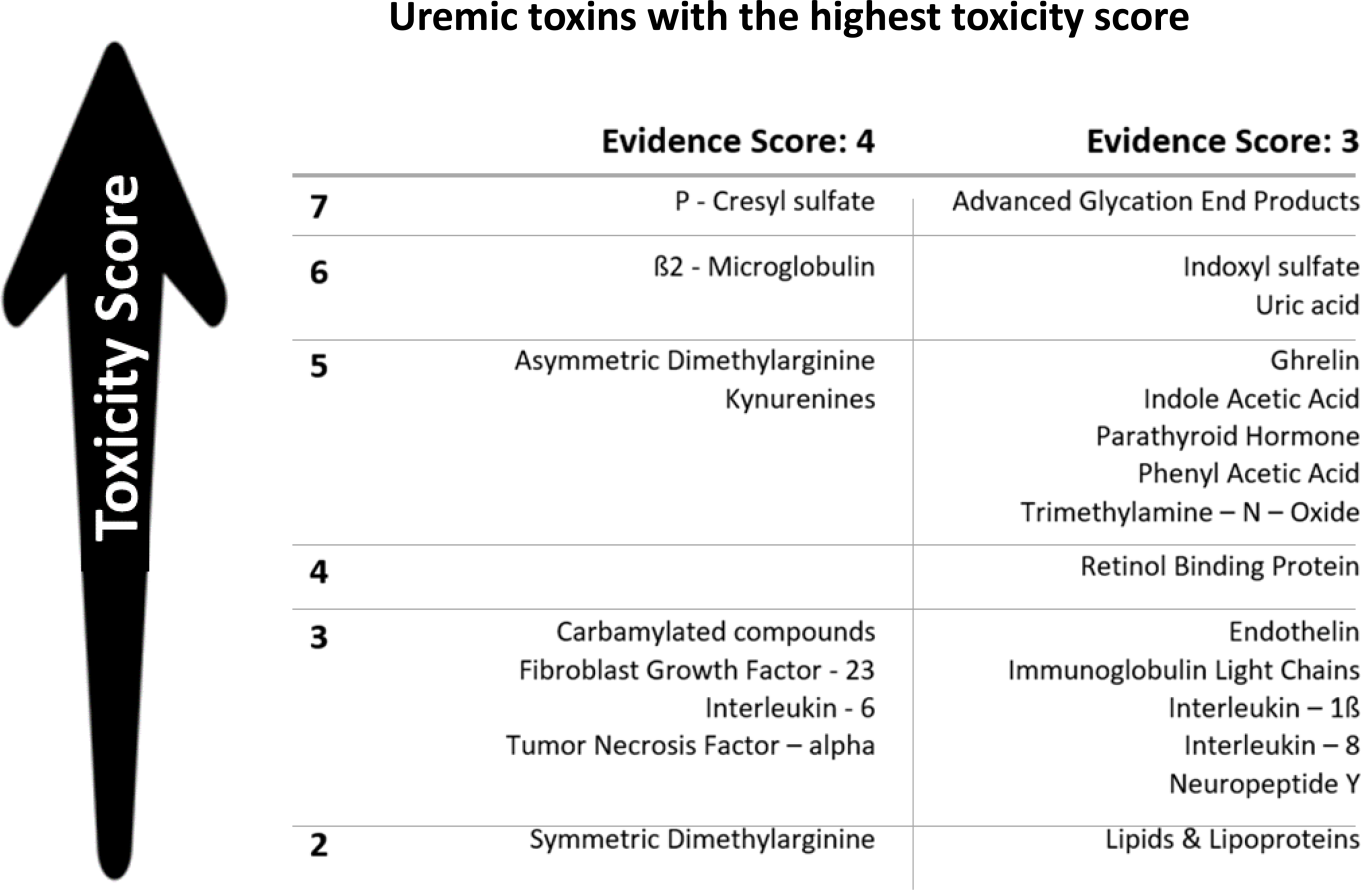

A systematic review approach was used to assess 71 uremic retention solutes. The score was based on clinical evidence (2 points) and experimental evidence (2 points) of detrimental effects, with a maximum of 4 points (summarized under “Evidence score”). A subclassification was applied based on the number of organ systems for which a negative impact was demonstrated (summarized under “Toxicity Score – arrow and number at left”). The highest-ranking toxins (scoring 4 and 3) are illustrated. The highest scores were obtained for a broad range of solutes (small water-soluble compounds, protein-bound compounds, middle molecules/small peptides, and large molecules whose size exceeds that of the pores of the kidney’s glomerular basement membranes). Modified from Vanholder et al (17).

Among the currently available renal replacement modalities, online hemodiafiltration (HDF) is considered the most advanced renal replacement therapy, offering improved clearance of larger uremic toxins and better hemodynamic tolerance (27). It differs from conventional MHD by infusing sterile fluid prepared ‘online’ from standard dialysis fluid into the bloodstream, enhancing blood cleansing (28), (29). Depending on convection volume, high-dosage HDF is associated with better survival rates compared to standard HD (29–32) with survival improving at about 55 to 75 l convection volume per week (29, 33). The CONVINCE trial provides evidence for the superiority of high-volume HDF with a 23% risk reduction in all-cause mortality (34). Mechanisms remain unclear but are likely multifactorial (35). The ongoing UK H4RT study is expected to further support HDF’s superiority over high-flux MHD (36, 37).

### 1.2 Systemic inflammation

Systemic inflammation is intrinsically linked to chronic kidney disease (CKD) (38), contributing to end-organ damage, particularly in the cardiovascular system and to premature ageing (39). In early CKD stages, over half of patients exhibit increased CRP levels (40), with severity escalating in later stages and complex inflammatory processes persisting in ESKD (41, 42). Uremic toxins contribute to pro-inflammatory effects, potentially catalysing the toxic uremic milieu, promoting risks for wasting and vascular disease (17, 37, 40, 43–46).

The large gap between the health span and lifespan of patients with chronic conditions is a major problem in modern healthcare (47). Lifestyle diseases contribute to senescence and vascular aging in CKD, with factors like uremic toxins, inflammation, and dialysis amplifying oxidative stress and inflammation (47, 48). Loss of gut microbiota biodiversity due to dietary effects further contributes to the allostatic load and aging (‘inflammaging’) (39). A five-year difference in biological and chronological age in patients requiring MHD emphasizes the need for interventions against uremic inflammation (47, 49), eg by reducing inflammatory molecule production and strategies enhancing their removal through improved dialytic clearance (40). Phenotyping according to comorbidities, clinical, social, or cultural and environmental stress factors that add to the global burden of CKD (50) and endotyping for uremic toxins by proteomics, metabolomics, and genetic markers based on selected biomarkers both aim to individualize HD strategies to improve patient outcomes (50–52).

Imbalances between oxidant and antioxidant production, or pro-inflammatory and anti-inflammatory mechanisms, mark the diseased state in CKD (53). Endogenous (*i.e.* endothelial nitric oxide synthase uncoupling) and exogenous mechanisms (*i.e.* environmental pollution) contribute to oxidative stress, impacting various organs and pathways. This results in increased proinflammatory cytokines, fuelling CKD progression in a vicious circle (54). Post-translational modifications (PTMs) are bidirectional links between oxidative stress, inflammation, and premature aging in CKD (55). PTMs, mainly phosphorylation, acetylation, and ubiquitination, can alter the structure and function of proteins, exerting adverse or beneficial effects on the kidneys (56, 57). Therapeutic approaches to limit oxidative stress and inflammation and to increase beneficial PTMs are available but need to be tested in clinical trials.

Systemic inflammation associated with chronic kidney disease is part of the uremic milieu disturbances that contribute to the morbidity and mortality of CKD patients. Besides senescence and aging, it is now well established that inflammation and its associated disorders contribute to accelerated cardiovascular disease (58–60), protein-energy wasting (61, 62), vascular calcification (63) and bone disorders, and loss of kidney function (50, 64).

### 1.3 Bioincompatibility and inflammation axis in MHD therapy

By reducing uremic toxin load and exposure, HD therapy helps to decrease inflammation. However, it introduces other factors, including bioincompatibility at the ECC-patient interface and dialysis fluid contamination, which can amplify inflammation (65–68). Bioincompatibility and inflammation are then inherent to all extracorporeal therapies representing an additional risk factor to MHD patients. Inflammation in MHD results from repetitive interactions between blood, the dialysis membrane, and contaminants in the dialysis fluid. This induces a series of cascade reactions, including complement activation, leukocyte activation, cytokine release, and the release of oxidative stress mediators, which sustain acute phase protein responses and contribute to related organ damage (10, 69). Mitigating bioincompatibility reactions by improving membrane design, circuit components, and dialysis fluid purity is crucial during MHD delivery, as any additional inflammation compounds the existing inflammatory burden of CKD. Despite advances in membrane, dialyzer, and extracorporeal circuit design, bioincompatibility remains a significant issue to address (70). Addressing these issues, e.g. by improving membrane and dialysate purity, helps ameliorate the detrimental effects of the uremic toxin milieu (2, 71).

Independent studies link increased inflammation to factors related to MHD, including ECC components, modality, and patient comorbidities (68). Addressing inflammation in MHD requires patient-individualized approaches due to its multifaceted origins (72)—factors like membrane type, modality, convective volume, and dialysis fluid quality impact MHD-induced inflammation (70). The CONTRAST study revealed that stable CRP values can be maintained over a 3-year online HDF treatment, while CRP levels increased during MHD (73). The use of ultrapure dialysis and sterile substitution fluids in HDF can further reduce inflammation by eliminating endotoxins from dialysis fluid (74) and help preserve residual kidney function in hemodialysis (75, 76).

### 1.4 Blood-material interactions: unavoidable consequences of maintenance HD

ECC components (needle, blood tubing, air-trap chamber, and dialyzer) interact with blood constituents like plasma proteins and blood cells (70). Without the natural endothelial lining protection, blood encounters polymeric materials and geometries in the dialysis circuit (77), triggering potent reactions and blood component activation. Altered plasma proteins or cells engage multiple biochemical pathways, and the patient’s intrinsic immune response and complement pathways are activated during blood-membrane interaction. Precise characterization and risk stratification guide membrane selection to reduce ECC-induced inflammation, though clinical studies on immune activation in dialysis patients remain scarce.

The 1970s discovery of cellulose-based MHD membranes causing high complement pathways and leukocyte activation sparked the biocompatibility debate. Synthetic membranes induce lower activation and less inflammatory response but don’t eliminate it. Despite membrane biocompatibility advancements, achieving entirely compatible membranes remains challenging (78). Biocompatibility extends beyond membranes to the rest of the ECC. Heparin, commonly used for anticoagulation, is effective but doesn’t fully prevent bioincompatibility or coagulation/complement-leukocyte activation (10), unlike citrate in specific extracorporeal therapies.

As an integral part of the innate immune system, complement plays a crucial role in antibody-mediated immunity (79). Its functions include defending against bacterial infection, bridging innate and adaptive immunity, and eliminating immune complexes. In chronic conditions like CKD, the complement system actively regulates various inflammatory responses. In HD, complement activation induces inflammation, promotes coagulation, and impairs host defense. Overactivation of complement pathways leads to aging-related diseases, e.g. Alzheimer’s disease or age-related macular degeneration (AMD). In CKD, the complement-inflammation axis mimics accelerated kidney aging, involving key players like Klotho expression, C1-inhibitor, pentraxin 3, pericytes, and endothelial-to-mesenchymal transition (74, 75, 80, 81). Understanding HD-induced inflammaging, causing immunological dysfunction and long-term complications that affect mortality (82), could inspire new therapeutic approaches to delay this ‘premature aging phenotype’ and kidney aging processes (83).

Minimizing adverse reactions requires comprehensive characterization of blood-contacting medical devices like dialyzers and membranes (84). Evaluating blood-membrane interactions demands sophisticated methodologies and intricate analytical techniques. Protein adsorption to biomaterials determines subsequent blood reactions, creating a biologically active surface (2-10 nm thick), of protein concentrations up to 1000 times higher than plasma. Proteomic technologies help characterize this protein adsorption/desorption (‘Vroman effect’) (85) creating a ‘blood-material interface’ that then mediates blood cell adhesion and coagulation or complement system activation (86). Platelet and leukocyte activation induce the release of extracellular vesicles, critical markers for cellular activation and intercellular communication (87). Those vesicles are a source of phosphatidylserine and tissue factor, amplifying thrombin generation (88). Flow cytometric techniques prove valuable for studying interactions of extracellular vesicles with biomaterials or with leukocyte subsets (89).

Dialyzer membranes vary in structure and biocompatibility, with different materials and production processes (90, 91). Even membranes from the same base polymer (*e.g.*, polysulfone) may exhibit different biocompatibility and safety profiles (92). Guided by clinical experience, manufacturers aim to minimize adverse reactions, balancing hydrophobic and hydrophilic properties to compromise between complement and coagulation pathway activation. Approaches like adding PVP or thrombomodulin to polysulfone aim to prevent coagulation or platelet adhesion, but data on their success are controversial (93). Vitamin E-coated membranes target oxidative stress and DNA damage reduction (94, 95), but their applicability is limited due to manufacturing constraints and costs.

ECC clotting issues in HD units are common, often stemming from variable and sub-optimal anticoagulation. Assessing clotting relies on subjective visual evaluations of dialyzer color (96). Objective methods such as micro-computed tomography scanning of fibers offer a superior approach to visual scoring, dry weight assessment, or pressure measurement for addressing anticoagulation and fiber patency issues, at least in experimental settings (97).

### 1.5 Advances in membrane and technology

Several symposium presentations focused on dialysis membrane and dialyzer development, highlighting engineering accomplishments in physiochemistry, thermodynamics, material sciences, and sterilization. The growing demand for dialyzer units necessitates mass production with robotic automation which requires a balance between productivity, recycling, environmental safety, and cost constraints. From conceptualization to delivery, attention is paid to uniformity, quality, clinical performance, and safety. Adjusting membrane ‘spinning’ parameters customizes pore size and porosity for desired sieving properties (98).

However, increasing porosity to remove larger uremic toxins may cause essential substance loss: albumin and amino acid leakage have to be kept to a minimum (19). Achieving optimal membrane structure relies on selecting core polymer–copolymer combinations with lower activation of biological pathways during blood-membrane interaction (98) and higher pro-inflammatory endotoxin retention (99), less protein fouling, and higher hydraulic and solute permeability (100). The development of superflux or super high-flux membranes, utilizing conventional polymers to broaden the range of uremic toxin removal, is a key focus of membrane research and advanced technology in dialysis. Medium Cutoff (MCO) and High Cutoff (HCO) membranes exemplify this research, aimed at improving the clearance of middle- and large-molecular-weight uremic toxins compared to traditional high-flux membranes (13, 101, 102). These advancements are achieved by increasing membrane pore size and enhancing internal filtration and backtransport mechanisms, which together optimize convective clearance. MCO membranes are specifically designed to remove middle- and large-sized uremic toxins while minimizing the loss of essential plasma proteins, such as albumin. MCO-based dialyzers are currently undergoing evaluation in clinical hemodialysis settings for their potential to enhance internal convective clearance, such as through internal hemodiafiltration. HCO membranes, with even larger pore sizes, enable the removal of larger molecules, including inflammatory mediators and nephrotoxic light chains, which are implicated in conditions like myeloma kidney. This technology holds promise for patients with acute kidney injury (AKI) characterized by severe inflammatory responses, as well as those requiring continuous kidney replacement therapy (CKRT). Despite the potential of these advanced membranes, clinical trials aimed at demonstrating their superiority have thus far yielded disappointing results. Further research is needed to fully assess their efficacy and optimize their clinical applications. Microporous silicone membranes, particularly silicon nanopore membranes (SNMs), are under development and evaluation for use in innovative wearable and implantable artificial kidney projects (103, 104). These membranes have uniform nanopores, typically ranging from 5 to 10 nanometers in size, which are critical for effectively filtering uremic toxins from the blood. However, the low porosity of these membranes (≤1%) presents a challenge. To address this, advances such as arrays of nanoslits are being explored to enhance permeability without compromising selectivity for small solutes. SNMs have shown promise in reducing the surface area needed for dialysis, which is a key factor in the miniaturization of artificial kidneys, potentially paving the way for the development of implantable devices. Trilayer interlinked graphene oxide membrane represents a cutting-edge advancement in dialysis technology, capitalizing on graphene’s unique properties to improve separation performance (105). Laboratory tests have demonstrated that these membranes can achieve high permeability and selectivity, offering potential advantages in biocompatibility and toxin removal efficiency compared to traditional polymeric membranes. These emerging membrane technologies present significant disruptive potential for enhancing renal replacement therapy. However, further work is needed in scaling up manufacturing processes and conducting outcome-based studies before these innovations can be fully integrated into the therapeutic arsenal of renal replacement therapy.

Innovative processes aimed at modifying membrane surface reactivity, such as stabilizing polyvinylpyrrolidone (PVP) on the blood-contacting surface, are crucial for reducing protein adsorption and enhancing biocompatibility (106): While conventional membranes elute PVP into the bloodstream (107), a recent approach stabilizes PVP with α-tocopherol (106). This modification creates a stable hydro layer at the inner surface, repelling excessive adsorption of proteins (107), enhancing biocompatibility, and maintaining high solute permeability throughout the treatment session (108).

Clinical evaluation and market introduction of innovations require evidence-based medicine (109). Real-world evidence is often used due to the time and cost challenges of interventional trials (110, 111). ECC and vascular access patency have been an issue (112), (113) that led to the exploration of alternative anticoagulation strategies, such as heparin-free MHD and membrane modification with fluorinated macromolecules (114). Dialyzer fibers present fluorinated end groups to create a surface that minimizes protein adsorption, platelet adhesion, and platelet activation. Evaluation of fluorinated macromolecule-modified dialyzers reveals promising results in reducing thrombus formation and bacterial adhesion (115), (116).

Efforts to improve HD membrane biocompatibility (117) historically relied on trial-and-error methods, often involving costly and time-intensive laboratory evaluations (118). A mathematical model based on morphology, chemistry, and interaction affinity of two membranes, along with *in vivo* and *in vitro* data (119), accurately predicted inflammatory biomarker release during HD therapy (120). Incorporating clinical operating conditions, the model´s applicability was extended to various membrane materials and facilitates the prediction and *in vitro* validation of inflammatory responses associated with synthetic membranes. The model offers a valuable tool to guide the development of novel materials and support evidence-based membrane synthesis (119, 120).

### 1.6 Emerging trends and future frontiers

Over decades, MHD operated by size-exclusion-based removal of uremic toxins across semipermeable membranes. Various avenues to improve HD strategies have been explored and presented at the symposium.

With a porous inner layer of polyethersulfone/polyvinylpyrrolidone (PES/PVP) (121) and an outer layer with activated carbon, Mixed Matrix fiber Membranes (MMMs) present a promising innovation (121–124) of high-flux membranes that showed limitations in protein-bound uremic toxin (PBUT) removal. MMMs offer effective filtration through diffusion, convection, and adsorption, facilitating MHD with reduced dialysate amounts. They provide high flux, low protein adsorption, low albumin leakage, and excellent hemocompatibility (115, 121), and may protect patients by adsorbing bacterial pyrogens from the dialysate (125). They also show potential for urea removal from dialysate (126) suggesting applications in portable artificial kidney systems.

**Figure 2:**
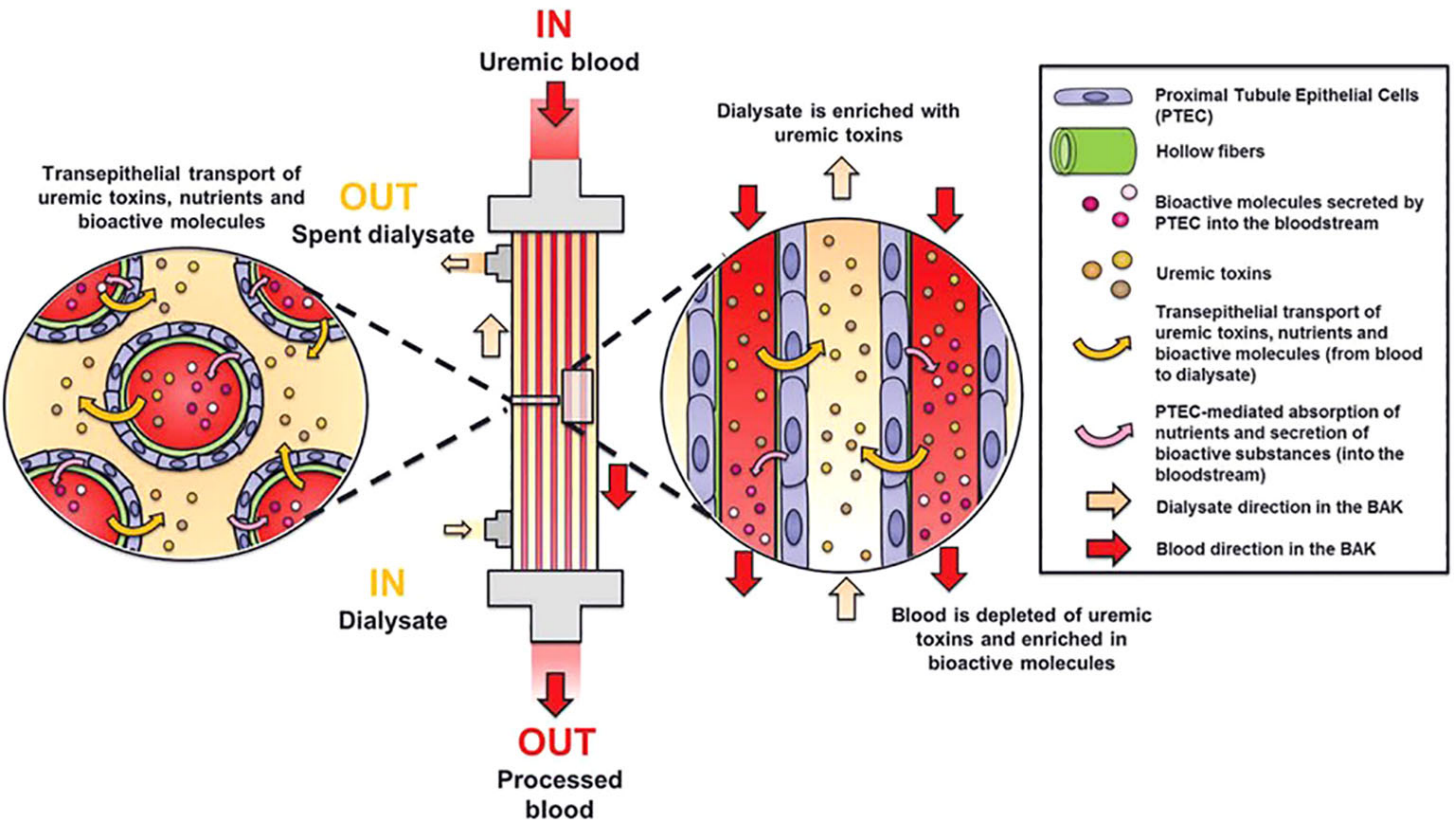
Principle of a bioartificial kidney. The living membrane of the BAK consists of human proximal tubule epithelial cells (PTEC) cultured on hollow membranes. These cells are capable of active transport of uremic toxins and nutrients and secretion of bioactive molecules. Modified from Van Gelder et al.(129).

Efforts to engineer cell-based kidney replacement therapy focus on creating a bioartificial kidney (BAK) replicating key functions of native kidneys, with uremic waste removal as a primary objective (127, 128). Human conditionally immortalized proximal tubular epithelial cell (ciPTEC) lines show promise for BAK development (**Figure 2**) (124), with the ability of active organic cation transport (130, 131), secretory clearance of albumin-bound uremic toxins and albumin reabsorption. Endocrine functions like secretion of the active form of vitamin D, auto-immune and inflammatory response, and tumorigenic and oncogenic effects have also been investigated (132–134). Absent alloo-stimulatory effects and ciPTEC monolayer stability indicate safety for BAK application, but challenges such as cell sourcing, organ scaffolding, and immune response must be addressed before clinical adaptation for human treatment (135, 136).

Several research groups are advancing both wearable and implantable artificial kidneys as viable alternatives to conventional MHD (105). Notable among these efforts is the KidneyX project in the US. Wearable artificial kidneys (WAKs) are designed to be lightweight and portable, offering continuous dialysis and enhanced patient mobility. Implantable artificial kidneys (IAKs) aim to replace failing kidneys through surgical implantation of miniaturized artificial kidney (e.g., silicone nanoporous slit membranes and advanced housing and connecting materials). These devices promise a more permanent and transformative solution. Despite significant technological advancements, challenges such as device size, biocompatibility, vascular access, bladder connection, toxin removal efficiency, and long-term functionality still need to be addressed through clinical trials. Additionally, recent progress in the transplantation of genetically modified porcine kidneys has shown promising results and is approaching clinical application (137, 138).

Artificial intelligence (AI) applications in nephrology, though scarce, show promise (139, 140). Deep learning, a subset of machine learning, involves multi-layered neural networks learning from extensive data (141). The AI application process uses problem definition, data preparation, model building, and data validation steps. In CKD care AI has been successfully applied to predict clinical events, to provide treatment decision aids for optimal drug prescription in anemia control, and to identify patterns for event development (*e.g.*, classification of phenotypical clusters of arteriovenous fistula aneurysms) (142). Optimized machine learning models could enhance risk identification and drive pre-emptive interventions (143). While AI in dialysis practice is in its infancy, more research is needed for consolidated aids in clinical decision-making, and future applications may offer real-time, continuous recommendations for optimal kidney care outcomes (144).

Emerging trends in HD address the growing demand for kidney replacement therapies and the limitations of the current provider system (145). Therapeutic advances were associated with parallel reductions in short-term risk of death and major adverse CV events (143). More frequent and longer MHD sessions show improvements in CV markers (146, 147), and flexible care models, including home or satellite HD and incremental, extended, and shared care, enhance treatment options (135, 148, 149). Wearable artificial kidneys aim for continuous blood purification and patient autonomy, but challenges like electrolyte balance (150) and suitable membrane selection (151) need to be addressed. Further investigation into preserving residual kidney function and modulating gut microbiota are exciting prospects for shaping the future of dialysis.

This future cannot be designed without considering the future of our planet. Dialysis therapies come at an enormous environmental cost due to their water consumption, greenhouse gas emissions, and waste production. Policymakers, manufacturers, providers, healthcare providers, and (future) patients need to put efforts into implementing urgent environmental changes which must include but are not limited to dialysate regeneration, dialysate flow reduction, water distillation systems for dialysate production, and biodegradable and bio-based polymers (150, 152).

## Data Availability

The original contributions presented in the study are included in the article/supplementary material. Further inquiries can be directed to the corresponding author.
